# Osteochondral tissue engineering in translational practice: histological assessments and scoring systems

**DOI:** 10.3389/fbioe.2024.1434323

**Published:** 2024-08-02

**Authors:** Mengying Cui, Yang Sun, Xiaoyang Zhang, Pengju Yang, Weibo Jiang

**Affiliations:** ^1^ The Second Hospital of Jilin University, Jilin, China; ^2^ Orthopedic Medical Center, The Second Hospital of Jilin University, Jilin, China

**Keywords:** osteochondral repair, tissue engineering, osteochondral grafts, histological assessment, histological scoring system

## Abstract

Osteochondral lesions are common pathological alterations in synovial joints. Different techniques have been designed to achieve osteochondral repair, and tissue-engineered osteochondral grafts have shown the most promise. Histological assessments and related scoring systems are crucial for evaluating the quality of regenerated tissue, and the interpretation and comparison of various repair techniques require the establishment of a reliable and widely accepted histological method. To date, there is still no consensus on the type of histological assessment and scoring system that should be used for osteochondral repair. In this review, we summarize common osteochondral staining methods, discuss the criteria regarding high-quality histological images, and assess the current histological scoring systems for osteochondral regeneration. Safranin O/Fast green is the most widely used staining method for the cartilage layer, whereas Gomori and Van Gieson staining detect new bone formation. We suggest including the graft–host interface and more sections together with the basic histological information for images. An ideal scoring system should analyze both the cartilage and bone regions, especially for the subchondral bone plate. Furthermore, histological assessments should be performed over a longer period of time to minimize discrepancies caused by defect size and animal species.

## 1 Introduction

The articular cartilage layer, the outermost portion of the osteochondral unit, provides tremendous durability and resilience, but limited repair capacity. In our joints, articular cartilage can reduce friction between articular bone and distribute various stress loading to underlying subchondral bone (Wang et al., 2021). The osteochondral unit, which consists of articular cartilage and underlies subchondral bone and the bone-cartilage interface, provides biomolecular and mechanical support to the upper cartilage layer. However, trauma-related injuries and natural wear within articular cartilage are difficult to repair and eventually lead to osteochondral lesions (Korthagen et al., 2019). To repair such lesions, various clinical strategies and techniques have been developed, such as arthroscopic debridement and lavage, marrow-stimulating techniques, osteochondral autografts or allografts, cell-implantation-based treatments, and total arthroplasty (Chen et al., 2019a; Ye et al., 2018). Unfortunately, these techniques have certain limitations during osteochondral repair and do not provide long-lasting satisfactory outcomes (Cho et al., 2020).

Osteochondral tissue engineering aims at creating cartilage-bone units or substitutes for repairing clinical osteochondral defects, and has successfully fabricated and utilized both bone and cartilage substitutes in clinical practice (Chen et al., 2018). It is generally accepted that osteochondral tissue engineering can provide better repair/regeneration than other defect-resurfacing methods. Tissue engineering can produce the appropriate shape, thickness, mechanical properties, surface integration, biocompatible properties, and self-healing and remodeling abilities, thereby showing promise for future clinical practices. These desired factors are interdependent and have been the focus of numerous osteochondral tissue engineering studies. However, to date, no consensus has been reached regarding the optimal tissue engineering design strategy for achieving satisfactory osteochondral repair (Zhu et al., 2017). An effort to combine different osteochondral graft designs and fabrication methods may provide better osteochondral repair and regeneration than other tissue-resurfacing methods.

Thus, to establish a reliable osteochondral repair method, it is necessary to compare different osteochondral tissue-engineered graft designs and fabrication methods. Currently, structural and functional analyses in translational animal models represents a standard approach to evaluating osteochondral regeneration. Successful osteochondral repair should regenerate the original structure, including the bone and cartilage regions, and subchondral bone plate. Histological assessments, micro-computed tomography, and MRI are common methods for assessing structural regeneration *in vivo* (Murata et al., 2022). Functional analysis, a purpose-oriented tissue-scale evaluation, focuses on the biomechanical properties and lubrication of the tissue-engineered block (Critchley et al., 2020). A satisfactory biomechanical result, such as proper stiffness and flexibility, should always mimic the mechanical properties of a natural osteochondral unit (Zhai et al., 2018). Although functional analysis determines the success of osteochondral repair, histological evaluations provide insights into repair mechanisms and accelerate the pace of “bench to bedside”.

To assess osteochondral repair, various staining methods are used, as they can reveal the anisotropy of osteochondral units. Selecting an appropriate staining method is crucial for evaluating lesions and histological scoring systems. For comparing osteochondral repair, quantitative histological scoring systems are more objective and reliable than qualitative histological images. The first scoring system for cartilage repair was established in 1986 by O’Driscoll and colleagues, who attempted to formulate a scoring system to evaluate the quality of newly formed cartilage (O'Driscoll et al., 1986). Later, O’Driscoll scoring systems were modified by the addition of a subchondral bone evaluation section to better assess osteochondral repair (Gan et al., 2019; Wu et al., 2020). Since then, numerous histological scoring systems, such as International Cartilage Repair Society (ICRS) (Xing et al., 2020), Pineda (Yucekul et al., 2017), Wakitani (Lu et al., 2018), and Sellers scoring (Lin et al., 2020), have been introduced. Although the ICRS developed a standardized scoring system for osteochondral repair, the lack of agreement regarding a unified histological evaluation system impedes comparison of different studies (Mainil-Varlet et al., 2003).

An ideal histological scoring system for osteochondral repair should consider both structural regeneration (e.g., the regeneration of subchondral bone, the subchondral bone plate, and an appropriately thick cartilage layer) and compositional restoration (e.g., restoration of the glycosaminoglycan (GAG) and collagen II gradient) of an osteochondral unit. However, no system exists that would cover all these aspects and thus provide a global view of repaired osteochondral tissue. To this end, modified scoring systems have been established, including the modified ICRS (which includes basal and lateral integration) and O’Driscoll (which includes subchondral bone) scoring systems (Oshima et al., 2019; Sun et al., 2018). Potential bias of these modified systems should be noted, as most of them remain to be validated. Some studies have verified the intra- and inter-observer reproducibility of traditional histological scoring systems; however, they have focused mostly on how to interpret histological images and convert findings into histological scores. Corresponding histological images with histological scores is difficult, and variations may impede comparisons between different studies. Thus, it is important to establish standard classifications of histological evaluation parameters or indices.

In this paper, we performed a literature review of histological assessments and scoring systems for *in vivo* osteochondral repair with tissue-engineered grafts. We reviewed histological scoring systems and histological images used in studies of translational osteochondral repair. Furthermore, we identified and analyzed common histological staining methods for osteochondral defects repaired by tissue engineering. Finally, we summarized current limitations and challenges associated with histological scoring systems for osteochondral repair to guide future translational studies toward clinical osteochondral regenerative medicine.

## 2 The osteochondral unit: architecture, composition, and function

**FIGURE 1:**
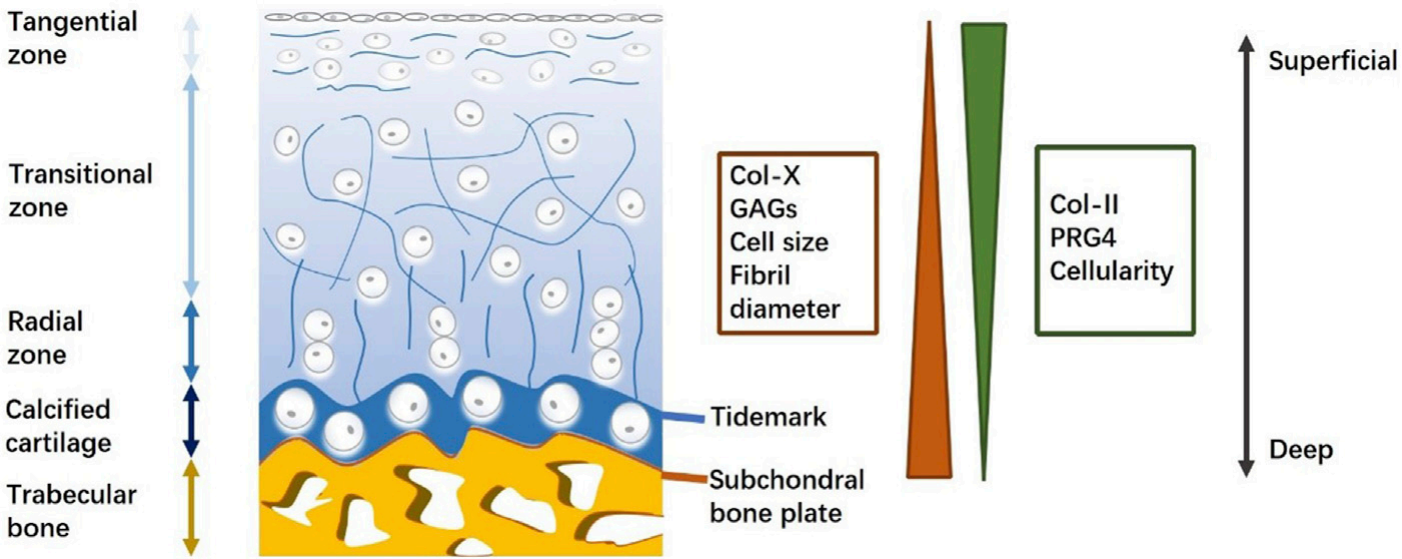
Schematic for osteochondral unit (structure and composition). Osteochondral structure and composition are varied with the depth from cartilage surface, displaying the heterogeneity of an osteochondral unit.

The osteochondral unit is an anisotropic tissue block with a hierarchical architecture that enables its loading and lubrication functions (Qiao et al., 2021). It consists of articular cartilage, subchondral bone plate, and trabecular bone from superficial to deep regions (Jia et al., 2018). These three distinct tissue types have completely different structures and compositions (Figure 1). Thus, different regions should be considered separately in the evaluation of osteochondral regeneration.

Articular cartilage, a precisely organized zonal tissue, covers the end of bones and protects joints by reducing friction between articular bone and distributing various stress loading to underlying subchondral bone (Cao et al., 2021). At the tissue level, articular cartilage thickness varies with location and local loading levels (Jia et al., 2018). At the cell level, chondrocytes that are embedded in the extracellular matrix (ECM) exhibit morphological differences across cartilage in correlation with depth (Boushell et al., 2019). At the molecular level, collagen fibers and proteoglycans weave into a network structure that provides resilience to stress, transports nutrients and signals, and enables chondrocyte adhesion (Zhu et al., 2018). Collagen II is the predominant component of collagen fibers and interacts with proteoglycans and other collagen types, including collagen IX, X, and XI (Wang et al., 2019). In contrast to collagen II, proteoglycan four expression decreases with depth, and collagen X and GAG cause adverse effects in the ECM (Hsieh et al., 2018). Collagen fibers cross-link with proteoglycan aggregates to protect cartilage against compressive and tensile forces (Wang et al., 2017). The arrangement of collagen fibers plays a significant role in the biomechanical properties of cartilage. Therefore, evaluating the orientation of regenerated collagen fibers is a promising approach to predicting the outcome of osteochondral repair.

Between the articular cartilage and subchondral bone, there exists a thin layer of calcified cartilage (Huang et al., 2021). This calcified cartilage is separated from non-calcified cartilage by a relatively irregular border known as the tidemark, which appears as a basophilic line on histological images (Zhang et al., 2017). Collagen fibers run through both the non-calcified and calcified zones. In this way, the tidemark enhances tensile strength and provides a physical barrier that protects distinct micro-environments in the cartilage and underlying bone.

The subchondral bone plate is a thin cortical lamella underlying the calcified cartilage zone that provides mechanical support for cartilage tissue. Compared with the compact structure of the subchondral bone plate, subchondral trabecular bone is a well-organized porous structure that includes osteocytes, osteoblasts, and osteoclasts (Sun et al., 2020). The vascularized and innervated trabecular zone exhibits potential endogenous healing capacity and feedback to biological and pathological conditions in the adjacent cartilage zone (Lin et al., 2020). Typical components of the trabecular zone are inorganic hydroxyapatites and organic collagen I, proteoglycans, and GAGs (Salonius et al., 2019). Proteoglycan composites, such as osteonectin, osteopontin, and osteocalcin, are specifically expressed in the trabecular bone.

The anisotropy of the osteochondral unit and the limited self-repair ability of articular cartilage make tissue engineering a promising technique for achieving osteochondral regeneration (Du et al., 2017). To date, many tissue-engineered osteochondral grafts have been evaluated in translational animal models (Kumai et al., 2019; Pérez-Silos et al., 2019; Gardner et al., 2019). However, an optimal design strategy that can achieve satisfactory osteochondral repair remains to be established. Thus, it is becoming increasingly important to establish a clear definition of successful osteochondral repair, and to compare results across different studies.

## 3 Methodology for osteochondral histological assessments

Histological evaluation is a widely accepted method for studying osteochondral regeneration, with the goal of analyzing regenerated tissue compositionally and structurally. The histochemical preparation of osteochondral tissue includes fixation, decalcification, processing, and cutting into sections (Chen et al., 2019b). For fixation and decalcification, osteochondral tissue should be treated differently from common connective tissues, as chondrocytes and ECM are prone to degradation by abundant fixation and decalcification agents. Thus, traditional decalcification agents (e.g., sulfuric, hydrochloric, and nitric acids) are unsuitable for preserving chondrocytes. Alternatively, organic acids and chelating agents avoid the aforementioned side effects and provide reliable decalcification. Ethylenediaminetetraacetic acid (EDTA), a chelating agent, is widely used in osteochondral tissue decalcification. When the decalcification is not urgent, EDTA is able to maintain the osteochondral tissue in a life-like state (Gong et al., 2020; Liu et al., 2017). Furthermore, for time-limited decalcification, formic acid is recommended. Fixed and decalcified tissue blocks should be embedded in paraffin to enhance their mechanical strength for further sectioning (Wang et al., 2019). Once cut, the tissue sections are fixed in warm solution, are placed on microscope slides, and undergo deparaffinization in xylene for upcoming staining (Murata et al., 2018).

Hematoxylin and eosin staining is a common method for observing pathological changes in cartilage. However, for the evaluation of cartilage repair, various other histological staining methods can be used (Li et al., 2023; Zhao et al., 2022). Currently, several histological criteria are considered to correlate with high-quality cartilage repair. For the chondral zone, the restoration of GAGs and proteoglycans (with an appropriate gradient), collagen II fibers (with an appropriate orientation and location), and chondrocytes (their morphology and quantity) should be evaluated in histological sections. Most *in vivo* osteochondral regeneration studies evaluate the presence of GAGs or proteoglycans with Safranin O and Alcian blue, positively charged dyes that selectively stain acid polysaccharides (Yu et al., 2022; Tamaddon et al., 2022). Safranin O and Alcian blue can stain carboxylates and sulfated polysaccharides at a pH of 2.5, whereas only Alcian blue stains more-acidic sulfated polysaccharides (e.g., with a pH of 1). Additionally, toluidine blue has been employed to stain GAGs (Cao et al., 2022). Although less commonly evaluated, collagen fibers are a vital component of cartilage and can be stained with Sirius Red (Burdis et al., 2022), Van Gieson (Cho et al., 2020), or Masson’s Trichrome (Lamparelli et al., 2021; Fang et al., 2022).

To histologically evaluate osteochondral regeneration in bone regions, Gomori, Van Gieson, and Alizarin Red staining are used. Gomori staining reveals bone alkaline phosphatase, a typical biomarker for osteoblasts (Yu et al., 2018). Alizarin Red stains calcium deposition in the osteo-zone, revealing new bone formation (Cunniffe et al., 2019). Although researchers prefer micro-computed tomography for the evaluation of bone reconstruction, this method does not reveal all details regarding osteochondral regeneration (Mendes et al., 2018; Nie et al., 2020). Histological sections not only reveal regenerated bone but also cartilage and the tidemark, thereby demonstrating any relationships between bone and cartilage repair (Table 1).

**TABLE 1:** Summary of staining methods for osteochondral tissue engineering.

Staining methods	Target component	Interpretation	Represent. tissue	Ref
HE	Chromatin, Extracellular matrix	Tissue structure	Cartilage, Bone	Nguyen et al. (2022)
Safranin-O/Fast green	Glycosaminoglycan	ECM quality	Cartilage	Mendes et al. (2020)
Alcian blue	Glycosaminoglycan	ECM quality	Cartilage	Filová et al. (2020)
Toluidine blue	Glycosaminoglycan	ECM quality	Cartilage	Yin et al. (2018)
Masson’s trichrome	Collagen II	Collagen content	Cartilage, Bone	Khanmohammadi et al. (2019)
Sirius red	Collagen I	Fiber alignment	Cartilage	Nie et al. (2019)
Alizarin red	Calcium	Calcium deposition	Bone	Cunniffe et al. (2019)
Goldner trichrome	Collagen, Trabecular bone	Bone formation	Cartilage, Bone	Asensio et al. (2021)
McNeal’s tetrachrome	Nucleus, Calcification	Newly formed Bone	Bone, Graft Residual	Shen et al. (2018)
Van gieson’s	Collagen	Distinguish Collagen and Muscle Fibers	Cartilage	Cho et al. (2020)
PAS	Glycosaminoglycan	ECM quality	Cartilage	Filová et al. (2020)
Mallory trichrome	Fibrous connective tissues	Fibrocartilage	Cartilage	Yucekul et al. (2017)
Sudan black	Residual polymers	Unbroken down Graft residuals	Graft residual	Gupta et al. (2017)
Gomori	Alkaline phosphatase	Osteoblast activity	Bone	Yu et al. (2018)
Tartrate-resistant acid phosphatase (TRAP)	TRAP	Multi-nucleated giant cells	Graft residual	Daly et al. (2018)
Diamidino-2-phenylindole (DAPI)	Aggrecan, collagen	ECM quality	Cartilage	Yucekul et al. (2017)
Alkaline phosphatase staining	Osteoblast	Osteochondral Unit	Bone-cartilage Interface	Nie et al. (2020)

As a complementary approach, immunohistochemical staining targets specific molecules in regenerated osteochondral tissue. Collagen I and II are two major molecules in immunohistochemical staining, as they are markers of fibrous and hyaline cartilage, respectively (Seong et al., 2017). During osteochondral repair, mechanically defective fibrous cartilage is undesirable, and thus higher collagen I expression indicates poor cartilage regeneration (Jiang et al., 2021). To date, several different markers related to articular cartilage regeneration have been evaluated. Aggrecan is the major structural component in articular cartilage (Yan et al., 2021). SOX-9, a transcription factor, plays a key role in chondrogenesis and can be detected by immunohistochemical methods (Qi et al., 2020; Lamparelli et al., 2022a). Furthermore, MMP-13 expression is less commonly evaluated, even though its increase may be related to poor-quality cartilage and chondrocytes (Liu et al., 2019). Very few studies have assessed subchondral bone regeneration with immunohistochemical methods. Nevertheless, collagen X and osteocalcin were shown to be markers of mineralized tissue, such as calcified cartilage and subchondral bone, in newly generated tissue (Daly et al., 2018; Ma et al., 2019).

## 4 Histological scoring systems for *in vivo* osteochondral regeneration

### 4.1 Overview of histological scoring systems

Evaluating the histological quality of osteochondral tissue is considered a reliable method to indicate osteochondral repair in translational animals. Numerous histological scoring systems were established to assess cartilage/osteochondral states in the field of cartilage tissue engineering. We found more than 20 histological scoring systems (13 original systems and their derivate systems) in studies on *in vivo* osteochondral regeneration (included in this review). Of these, ICRS, O’Driscoll, Wakitani, and their modified systems are most popular for evaluating *in vivo* osteochondral regeneration.

Nevertheless, there are still controversies regarding the selection of a histological scoring system. First, scoring systems designed with different purposes were used in the studies we reviewed, and the osteoarthritis histological system may yield unreliable results when evaluating osteochondral regeneration (Liu et al., 2023). Second, most histological scoring systems aim to assess cartilage regeneration (Tani et al., 2017; Duan et al., 2019); however, the quality of newly formed subchondral bone should be considered when assessing osteochondral unit regeneration. To fill this gap, investigators modified previous scoring systems by adding a bone-evaluating section (Ruvinov et al., 2019). However, large disparities between modified scoring systems exist and further impede comparisons between different tissue-engineering strategies (Jiang et al., 2018; Wang et al., 2018). Third, only a small number of scoring systems were validated by assessing reproducibility, sensitivity, and specificity (Duan et al., 2019).

**FIGURE 2:**
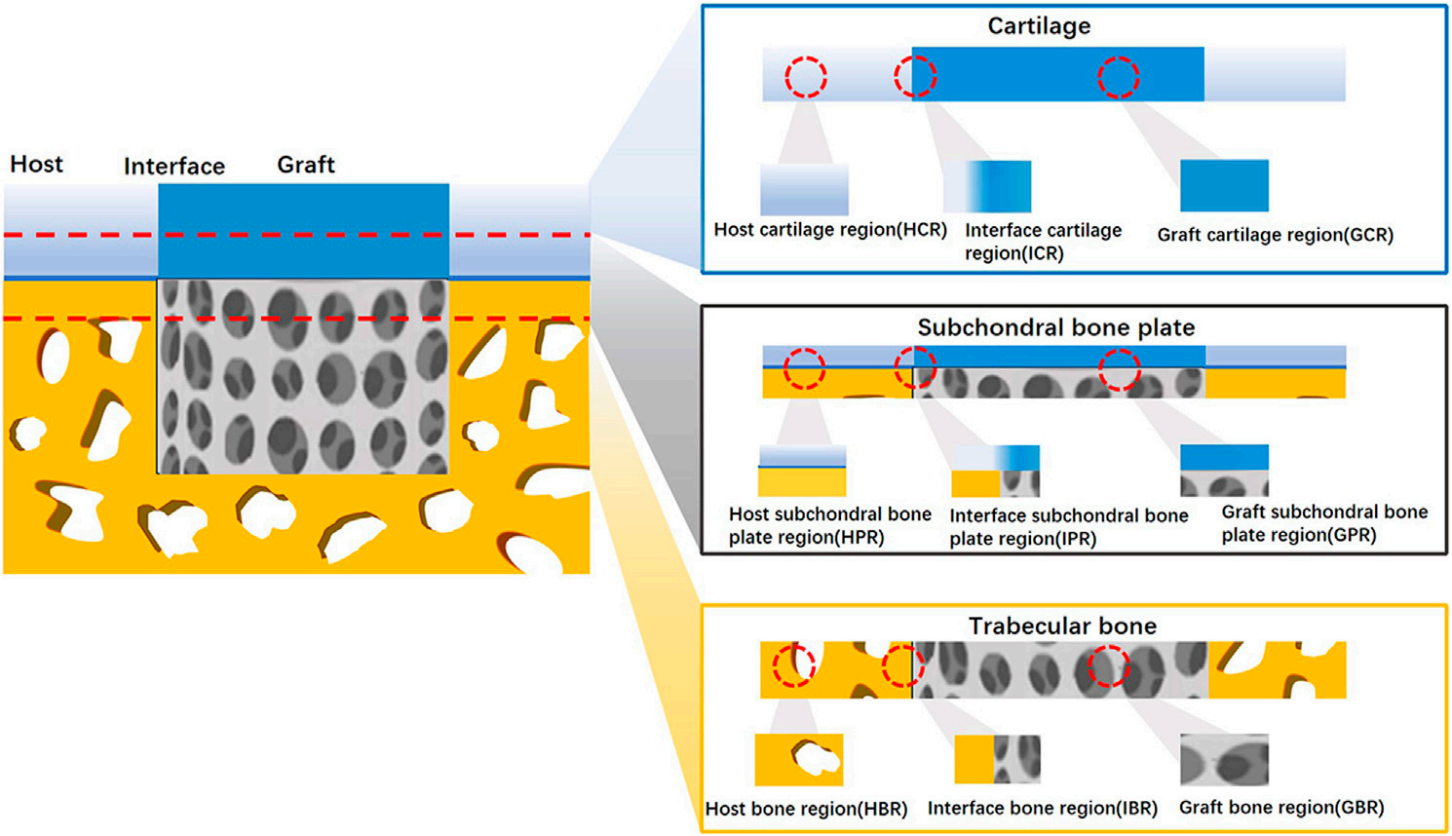
Spatial schematic for nine division osteochondral defect A regenerated osteochondral tissue block was divided into nine regions according to host–graft interface and cartilage-bone variation.

In theory, a complex scoring system with more parameters can provide comprehensive details and yield higher sensitivity. In fact, both regenerated cartilage and bone tissue should be evaluated, as unequal numbers of cartilage and bone parameters may cause bias and misunderstandings when repair results are interpreted through histological scores. Thus, selecting and organizing osteochondral repair parameters for different tissue regions may reduce the potential biases of current histological scoring systems. A typical osteochondral unit consists of cartilage, trabecular bone, and a subchondral bone plate. Upon implantation of a tissue-engineered osteochondral graft into a defect, the local area is divided into a graft zone, a host zone, and a graft–host interface zone. In this way, an osteochondral regeneration joint model can be divided into nine regions: the graft cartilage, graft subchondral bone plate, graft trabecular bone, host cartilage, host subchondral bone plate, host trabecular bone, cartilage interface, subchondral bone plate interface, and trabecular bone interface regions (Figure 2). According to these spatial subdivisions for osteochondral regeneration, all reported histological scoring system parameters will be reviewed and summarized.

Among the nine regions of osteochondral regeneration, the graft cartilage region has attracted the most attention. Unlike the graft region, host region parameters are seldom included in histological scoring systems. Conversely, higher evaluation rates were found in the cartilage, subchondral bone plate, and trabecular bone region. None of the studies included in this review used a histological scoring system to evaluate the host subchondral bone plate.

### 4.2 Region-specific osteochondral histological evaluations

#### 4.2.1 The graft cartilage region

Successful functional reconstruction of a joint should consist of structural and compositional cartilage regeneration. Thus, among the aforementioned nine regions, the graft cartilage region receives the most attention during osteochondral repair (Ding et al., 2022). The parameters for evaluating cartilage repair are similar across various histological scoring systems. At the tissue level, surface roughness (Chen et al., 2022; Wang et al., 2022), cartilage thickness (Zhang et al., 2022; Yan et al., 2020a), and ECM components are the most popular parameters for evaluating graft cartilage and are included in almost all scoring systems (Luo et al., 2022; Bozkurt et al., 2019). The surface of cartilage appears in a linear form that is easy to compare between slices. However, the omission of tiny fissures may be an inherent limitation in selecting sections. Thus, slides should be double-checked macroscopically (Zhang et al., 2018). Cartilage thickness is evaluated by comparisons with adjacent healthy cartilage; variable tissue thickness can negatively affect biomechanical structure and thereby cause osteochondral graft failure (Pascual-Garrido et al., 2019). In some cases, normal cartilage thickness in the graft region may not indicate high-quality osteochondral regeneration because the height of the newly formed subchondral bone plate may not be appropriate. This in turn may cause the overlying articular cartilage to be too high or low (Park et al., 2019).

ECM components surround and are produced by nearby chondrocytes, and indicate the cells’ status (Yin et al., 2018). ECM components are assessed in all the histological scoring systems that we reviewed; however, different scoring systems assess different ECM components. For example, the O’Driscoll (Widhiyanto et al., 2020) and Sellers (Lin et al., 2020) scoring systems evaluate GAGs by Safranin-O staining, whereas ICRS scoring systems evaluate GAGs by toluidine blue staining (Wang et al., 2018). As an alternative parameter, some histological scoring systems evaluate the percentage of osteochondral defect fulfillment, which is sensitive to relatively low-quality osteochondral repair with obvious defects (Petrovova et al., 2021).

At the cell level, the size, morphology (Shen et al., 2021), quantity (Mohan et al., 2017), and arrangement of chondrocytes (Wang et al., 2017) are widely used parameters in current histological scoring systems. The O’Driscoll scoring system tends to provide more details about chondrocytes and covers all the abovementioned parameters except cellularity (Cai et al., 2022). Most traditional histological scoring systems focus on cell morphology to classify cartilage, distinguishing between hyaline and fibrous cartilage. Recently, various modified ICRS and O’Driscoll scoring systems have taken chondrocyte clustering into account (Zhao et al., 2022; Yan et al., 2021). Chondrocyte clustering is an abnormal cell arrangement that correlates with immature or degenerated cartilage tissue (Xu et al., 2021). Chondrocytes and the ECM represent important aspects for histologically evaluating the graft cartilage region. During osteochondral regeneration, chondrocytes and the ECM always interact with each other, and tissue-engineering strategies attempt to influence this interaction and promote hyaline cartilage formation. Similar parameters across the various scoring systems demonstrate the consensus on histologically evaluating the graft cartilage region.

#### 4.2.2 The cartilage interface region

Because of the intrinsic avascular and denervated properties of articular cartilage, the cartilage interface between the graft and host is the most difficult region to reconstruct (Yang et al., 2018). Insufficiently repaired chondral interface tissue is still a challenge for the translation of osteochondral repair (Nordberg et al., 2021). Histologically, successful cartilage interface regeneration should exhibit continuous ECM in both the superficial layer (with a smooth surface) and the deep layer (without gaps) (Wei et al., 2019). ECM staining that is similar to an extent to that of the adjacent host cartilage region is also needed (Xing et al., 2021). Currently, two parameters related to the cartilage interface region are included in histological scoring systems. O’Driscoll, Wakitani, Sellers, ICRS, Fortier, and some modified scoring systems have assessed graft–host cartilage integration by checking gaps or lacks of continuity on either side of the graft (Xiao et al., 2019; Kumbhar et al., 2017). Conversely, to determine cartilage interface quality, some modified ICRS scoring systems classify the tissue into fibrous and cartilage-like tissue (Wong et al., 2018). Both “tissue continuity” and “cartilage-like” characteristics are parameters that focus on ECM status. Because of the limited space between tissue-engineered grafts and host cartilage tissue, chondrocyte-related parameters are not evaluated in this region. Although parameters related to the cartilage interface region are limited, the regeneration of the cartilage interface is crucial and should never be ignored during histological evaluation.

#### 4.2.3 The host cartilage region

Although osteochondral regeneration does not occur within the host cartilage region, ongoing regeneration affects adjacent healthy cartilage and causes further changes. In turn, even if successful osteochondral tissue is achieved, an impaired host cartilage region may cause the new osteochondral tissue block to rapidly break down. Quite a few scoring systems have taken the host cartilage region into account. The O’Driscoll scoring system analyzes chondrocyte cellularity, cluster changes, and ECM staining to determine host cartilage quality (Kim et al., 2021). Other modified scoring systems evaluate the cellularity and GAG content of adjacent host cartilage (Yan et al., 2021). Along with the cartilage tissue around the graft, the cartilage on the opposite side of a joint should also be assessed because it plays a role in sliding and loading with the graft cartilage region. However, few histological scoring systems currently evaluate this region. With the development of osteochondral tissue engineering, we believe that the host cartilage region will become another meaningful zone to analyze and record. A modified histological scoring system that includes the host cartilage region will provide more comprehensive details. Enhancing histological evaluations to include not only the defect but the entire joint will advance the translational progression of osteochondral tissue engineering.

#### 4.2.4 The graft bone region

The graft bone region has seldom been evaluated in previous studies on cartilage or osteochondral repair. Two reasons account for the unbalanced evaluation of bone and cartilage. First, compared with cartilage tissue, bone tissue is easier to regenerate, as it contains abundant blood vessels and multi-potential cells (Shang et al., 2020). Second, to some extent, the quality of cartilage repair correlates with the quality of underlying bone repair (Filová et al., 2020). Therefore, many studies still use simple histological evaluations of cartilage. Recently, the importance of bone regeneration in osteochondral defects was demonstrated, leading to the development of suitable histological scoring systems for evaluating bone regeneration, especially the graft bone region (Dai et al., 2018). The primary parameter for evaluating bone regeneration is “defect fulfillment” in the graft bone region. Among the traditional scoring systems (Petrovova et al., 2021), the Sellers system measures the percentage of new subchondral bone, i.e., defect fulfillment (Lin et al., 2020). The modified ICRS (Fang et al., 2022) and O’Driscoll scoring systems (Cho et al., 2020) and some new systems have included this parameter for preliminary evaluations. Considering specific trabecular bone architecture, some modified scoring systems try to analyze bone tissue morphology. Most bone morphology evaluating systems are derived from the Wakitani system and also include the “bone defect fulfillment” parameter (Wang et al., 2019). Another modified ICRS scoring system also estimates bone morphology but without “bone defect fulfillment”, which may cause a relatively lower proportion of evaluations related to bone tissue (Oshima et al., 2019).

Furthermore, “inflammation” is another parameter for osteochondral repair, as exogenous material implanted in the local defect may induce rejection by the host. Although biocompatibility has already been tested *ex vivo*, host–graft reactions should be further investigated. Accordingly, some researchers have added an “inflammation” parameter to the O’Driscoll scoring system (Jiang et al., 2021). Our review demonstrates that graft bone regions are evaluated less often than graft cartilage regions. However, future studies should discuss and determine the degrees to which regions of graft bone *versus* cartilage should be analyzed in histological scoring systems.

To promote subchondral bone regeneration, tissue-engineering materials, such as hydroxyapatite, tricalcium phosphate, decalcified bone matrix, bioactive glass, and metal materials, can be incorporated into the bone region to enhance the graft’s mechanical strength. However, this may impede the formation of new subchondral bone (Zhang et al., 2021). Some semi-quantitative histological scoring systems have analyzed the “percentage degradation of the implant” during rabbit osteochondral repair (Cho et al., 2020). Material degradation parameters are specifically appropriate for assessments of grafts, especially graft bone regions. An optimal time point of graft degradation and new bone formation is desirable.

#### 4.2.5 The interface bone and host bone region

The modified O’Driscoll scoring system is one of the few systems that analyzes the integration of the osteochondral graft with its surroundings and is thus adequate for evaluating bone interface regeneration (Du et al., 2017). Similar to the parameter of cartilage integration, the parameter “extent of new tissue bonding with adjacent bone” includes the following subitems: “complete on both edges”, “complete on one edge”, “partial on both edges”, and “without continuity on either edge”. Besides lateral integration, basal integration between the osteochondral graft and host bone tissue should be adequate. Thus, a small number of parameters were chosen for evaluating the basal interface. OsScore and ICRS II have assessed the basal integration of cartilage regeneration (Stefani et al., 2020; Bianchi et al., 2019). This review includes only four studies with modified systems that evaluate the host bone region. It seems that researchers, rather than using quantitative parameters, prefer to subjectively assess the host bone during osteochondral repair (Khanmohammadi et al., 2019; Mohan et al., 2017).

#### 4.2.6 The graft subchondral bone plate region

The subchondral bone plate, a previously underestimated factor, influences the long-term performance of osteochondral regeneration. Previous systems seldom focused on subchondral bone plate regeneration, and a few systems analyze the osteochondral junction and tidemark reconstruction instead (Liao et al., 2017). The Pineda scoring system evaluates the reconstruction of the osteochondral junction with simple classifications: “complete reconstruction”, “almost reconstruction”, and “not close”. The Sellers system indirectly assesses the formation of new subchondral bone by measuring the extent of tidemark formation and using the following classifications: complete, 75%–99%, 50%–74%, 25%–49%, and <25% (Lin et al., 2020). A modified version of the O’Driscoll system indirectly evaluates the subchondral bone plate with the parameter “bonding of repair cartilage to *de novo* subchondral bone” (Du et al., 2017). However, the extent of tidemark formation is not equally representative of subchondral bone plate regeneration. Even if subchondral bone plate regeneration is successful, heterotopic osteophytes and subchondral bone protrusion into the cartilage layer may corrupt the tidemark (Feng et al., 2020a). The thickness of a newly formed subchondral bone plate is closely correlated with its mechanical properties. As such, the subchondral bone plate should be restored to a similar thickness to that of the adjacent subchondral bone plate. However, no parameter related to subchondral bone plate thickness has been used in studies on translational tissue engineering. As the increasingly predominant role of the subchondral bone plate is being recognized, more related parameters should be included in histological assessments of osteochondral regeneration.

#### 4.2.7 The interface and host subchondral bone plate regions

Current histological scoring systems have not been used to evaluate subchondral bone plate interface formation or the host subchondral bone plate region. For multilayer or gradient tissue-engineered osteochondral grafts, specific layers or regions were designed to correspond with osteochondral bone plates (Gupta et al., 2017; Idaszek et al., 2019). These specific layers or regions have potential to transform into new osteochondral bone plates *in situ*. Conversely, typical elementary tissue-engineered one- or two-layered osteochondral grafts display a transitional regeneration process of subchondral bone plate from bottom to top and finally approach the host subchondral bone plate (Feng et al., 2020b; Liu et al., 2020). Therefore, plate integration is an important sign of completed osteochondral repair.

**FIGURE 3:**
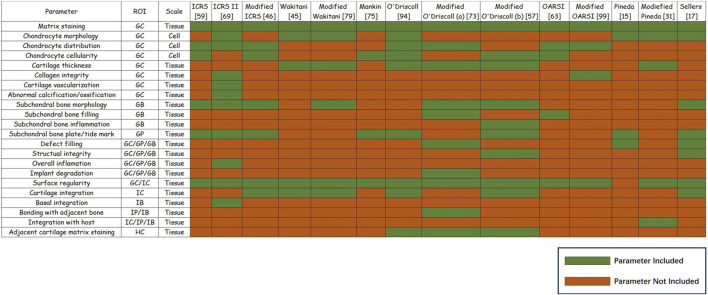
Summary of histological parameters for osteochondral regeneration. Summary of histological parameters for osteochondral regeneration. Parameters’ region of interest (ROI) and scale are shown. For the most widely used 14 histological scoring systems for osteochondral regeneration, the green boxes refer to specific parameters that were included in this system, while red boxes indicate that the parameter was not included in this system.

Osteochondral regeneration should also be spatially evaluated. In other words, assessments of the quality of tissue repair should also consider the relationships between different regions. Traditional histological scoring systems for osteochondral repair are result-oriented, focusing on the quality of repaired cartilage and thus potentially ignoring any mechanisms underlying osteochondral regeneration failure. For tissue-engineered grafts, assessments that determine the causes of failure are preferred. To this end, a spatially based histological scoring system shows promise. Current histological scoring system parameters were divided into nine separate regions, and the ratio of every single region in histological scoring systems and current or potential supplementary parameters were summarized (Figure 3).

### 4.3 Validation of histological scoring systems

Validating histological scoring systems to improve the reliability of observations and the comparability between studies is widely accepted (Rutgers et al., 2010). However, only a few scoring systems, such as the O’Driscoll and Pineda systems, have been validated (Moojen et al., 2002). A characteristic feature of healthy cartilage integrity is the content of proteoglycans, essential biochemical factors, which have been correlated with the O’Driscoll and Pineda systems for validation (Grogan et al., 2006). Nevertheless, the reproducibility of most histological scoring systems has not been evaluated or validated to date. To validate a scoring system, the system is usually compared with already validated macro- or micro-scoring systems, biochemical factors, or other validated evaluation methods (Custers et al., 2007). Nevertheless, it is still unclear whether a histological score can be correlated with the quantity of biomolecules in repaired tissue (Orth et al., 2012a).

### 4.4 A temporal scale for osteochondral histological evaluation

Osteochondral regeneration usually proceeds in specific spatial and chronological patterns. However, histological evaluations for translational studies are limited by the test frequency and ignore temporal alterations of cartilage and subchondral bone compartments. An adequate experimental duration and proper observation frequency should be emphasized. Most *in vivo* osteochondral regeneration studies share an observational time span of 1–52 weeks. Additionally, more than half of *in vivo* osteochondral regeneration studies histologically evaluated regeneration only once.

Assessing the histological performance of blank groups is another potential way to compensate the drawbacks of temporal histological evaluations. The histological performance of blank groups, especially from similar *in vivo* osteochondral repair studies, may contribute to our understanding of the chronology of osteochondral self-repair patterns (Orth et al., 2012a). Osteochondral repair can be compared between similar species, ages, genders, defect locations, and defect sizes. Although this review includes a limited number of studies, a trend can be observed in which regeneration starts from the bottom and lateral side of osteochondral defects.

## 5 Determining the quality of histological images

High-quality histological images not only provide comprehensive information regarding repair but also indicate the quality of the research to some extent. Although histological assessment is a widely acknowledged routine evaluation for osteochondral repair, the quality of histological images varies across studies. The basic information that should be included in histological images is as follows. First, an ideal histological image should contain clear scale bars and display integrated regions of interest (Yan et al., 2020b). The term integrated tissue regions refers to the graft–host interface and relevant adjacent native tissues. As there are two lateral boundaries for cylindrical osteochondral defects, neither interface should be ignored. Indicators that illuminate the graft contour or interface are recommended, as they enable more precise interpretation, especially for images that only display partial defect regions (Tani et al., 2018; He et al., 2017). Second, the image’s orientation, e.g., anterior/posterior and medial/lateral, serves as meaningful information to correlate micro-features with macroscopical appearance (Nie et al., 2020). Moreover, section location on a joint is another way to facilitate the micro-to-macro correlation (Salonius et al., 2019). Third, histological sections for various staining should be selected from an adjacent location. Some studies even attempt to correspond histological images with micro-computed tomography results, which yields more details (Liu et al., 2020; Fu et al., 2016).

**FIGURE 4:**
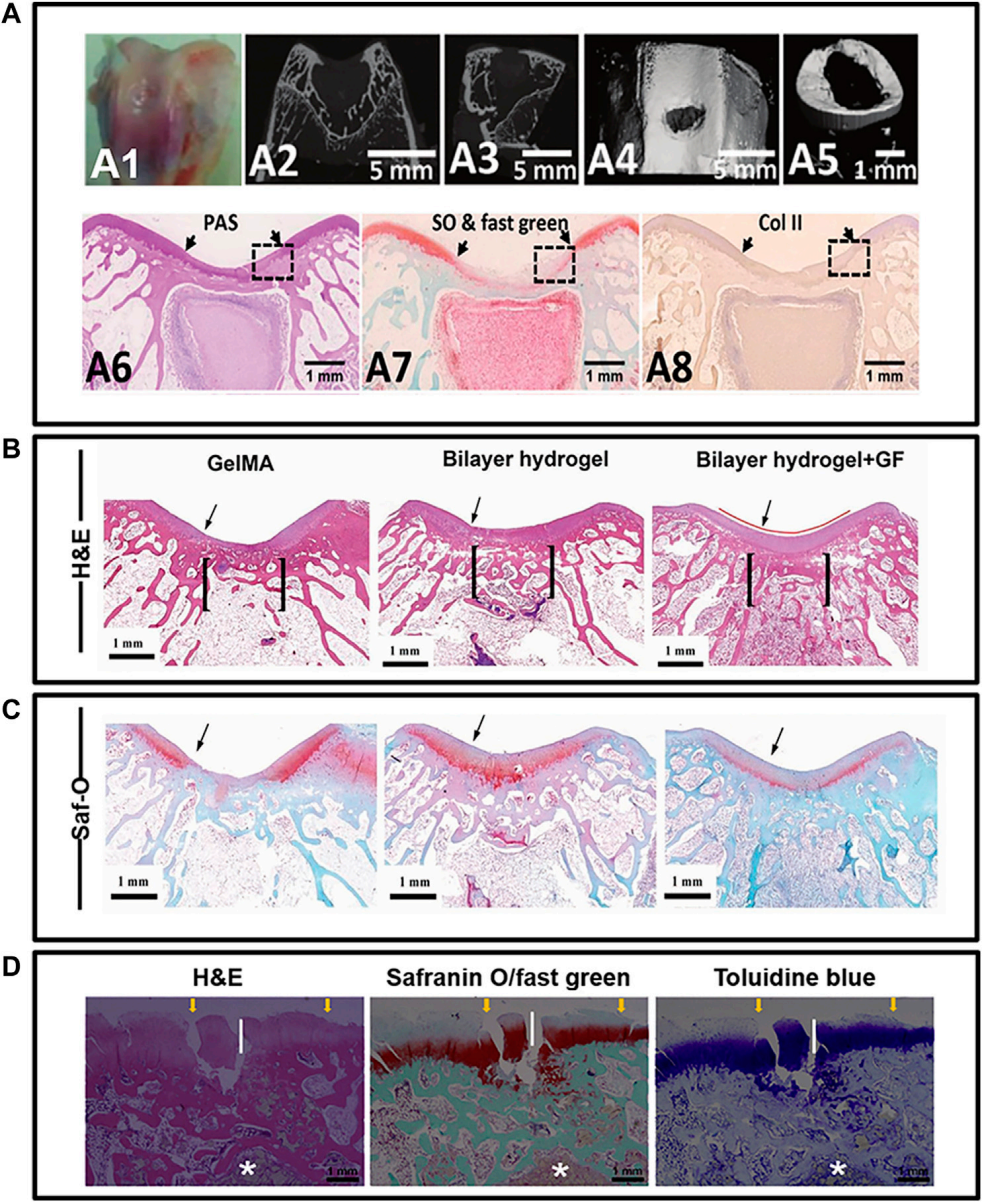
Principles for histological image quality evaluation. **(A)** pictures illustrated the osteochondral regeneration with silk fibroin/collagen scaffold in New Zealand Rabbits. Host-graft interface was clearly displayed by two distinct arrows. Besides, the orientation of histological image was obviously provided by corresponding to CT images. It is also recommended to place different stained images (A6, A7, A8) from similar location. Unfortunately, these pictures do not include all the region of interest for osteochondral graft. **(B, C)** pictures provide the osteochondral regeneration outcomes in rabbits model, with mussel-inspired hydrogel. They utilized columnar indicator to show the intact region of interest and graft contour for interpreting the regeneration. The *in vivo* osteochondral regeneration using a bi-layered poly(lactide-coglycolide) porous scaffold is show in **(D)** pictures. For the short-term repair results, asterisk was used to indicate the graft residuals. Transplants alternation and interaction with host over time could be better interpreted. Such residues indicator contributed to a high-quality image and thus desired. Although all histological images involved in this figure are harvested from rabbits, overall appearance of **(D)** is totally different from **(A–C)** which caused by the sample sectioning. Therefore, histological images orientation, such as medial, lateral, anterior and posterior, is recommended to improve clarity and consistent of future histological display. *(A) was cited from ref Feng et al. (2020b), **(B, C)** was cited from ref Gan et al. (2019), and **(D)** was cited from ref Duan et al. (2019). And we have got copyright permission from “Royal Society of Chemistry”, “WILEY”, and “ELSEVIER”.

Conversely, for osteochondral regeneration, muti-layer grafts exhibit the original location of each layer on histological images, revealing early pathological changes in each layer with different components (Nordberg et al., 2021). Additionally, graft residues should also be recorded and marked during microscopy (Zhou et al., 2017). Histological images for evaluating osteochondral repair should be compared with other examinations. Comparing images from different staining methods, radiological examinations, and macroscopic analyses (both at the level of the graft and joint) constitute the standard of a high-quality histological evaluation (Duan et al., 2019; Gan et al., 2019; Feng et al., 2020b) (Figure 4).

## 6 Current limitations and potential improvements of osteochondral histological evaluation

Histological evaluations are important for determining osteochondral regeneration in translational animal models. In this review, we summarized current histological methodologies and scoring systems for osteochondral tissue engineering. Currently, researchers focus on achieving acceptable osteochondral regeneration. However, there is no consensus on how to properly and objectively assess osteochondral regeneration, which may impede the progression of osteochondral tissue engineering. Any limitations and challenges of current histological scoring systems affect future translational research.

One major drawback of current histological scoring systems is the lack of consistent standards for the evaluation of bone. For the reconstruction of a smooth articular surface, previous studies focused on the regeneration of the cartilage layer, which was analyzed by the O’Driscoll (Wang et al., 2022), Wakitani (Zhang et al., 2022), and Mankin scoring systems (Peng et al., 2019). These scoring systems provide widely acknowledged parameters for cartilage repair, from compositional to structural reconstruction. However, parameters regarding the bone region are not included in these systems. With the growing recognition of the importance of subchondral bone during osteochondral regeneration, histological scoring systems have been supplemented with additional parameters, such as subchondral bone plate reconstruction and trabecular bone morphology and fulfillments (Cho et al., 2020). Nevertheless, parameters regarding the subchondral bone plate and subchondral trabecular bone still share only a small part of the entire scoring system. Such systems may underestimate the function of bone during the repair of osteochondral units. Although CT provides details of bone tissue, our still-lacking understanding of the relationships between subchondral bone and overlying cartilage on CT images limits interpretations regarding inadequate cartilage regeneration (Gu et al., 2022). Therefore, future histological scoring systems that place more importance on subchondral bone, especially the subchondral bone plate, are necessary.

In addition to the reconstruction of the subchondral bone plate, the height and integration of the subchondral bone plate have also been suggested to be included in osteochondral scoring systems. Bone plate heights of self-healing osteochondral defects vary among animal species and defect types; however, a height similar to that of the host subchondral bone plate is ideal (Orth et al., 2012b). Both subchondral bone that protrudes into cartilage and a concaved subchondral bone plate impair the long-term performance of tissue-engineered osteochondral grafts.

Current histological scoring systems indicate a trend of evaluating regeneration at the joint level rather than the defect level. Along with this transition, several systems have started to consider the host region as well (Cai et al., 2022). It is now encouraged to histologically evaluate host bone and synovium to determine the effect of heterogenous tissue-engineered grafts on surrounding micro-environments. Nevertheless, current scoring systems seldom include these host-related parameters, which may be due to overlaps with *in vitro* biocompatibility studies (Tian et al., 2021; Lamparelli et al., 2022b). For tissue-engineered osteochondral grafts, any graft residues should also be recorded, as the occupying effect and degradation products will influence local regeneration (Ran et al., 2021).

**FIGURE 5:**
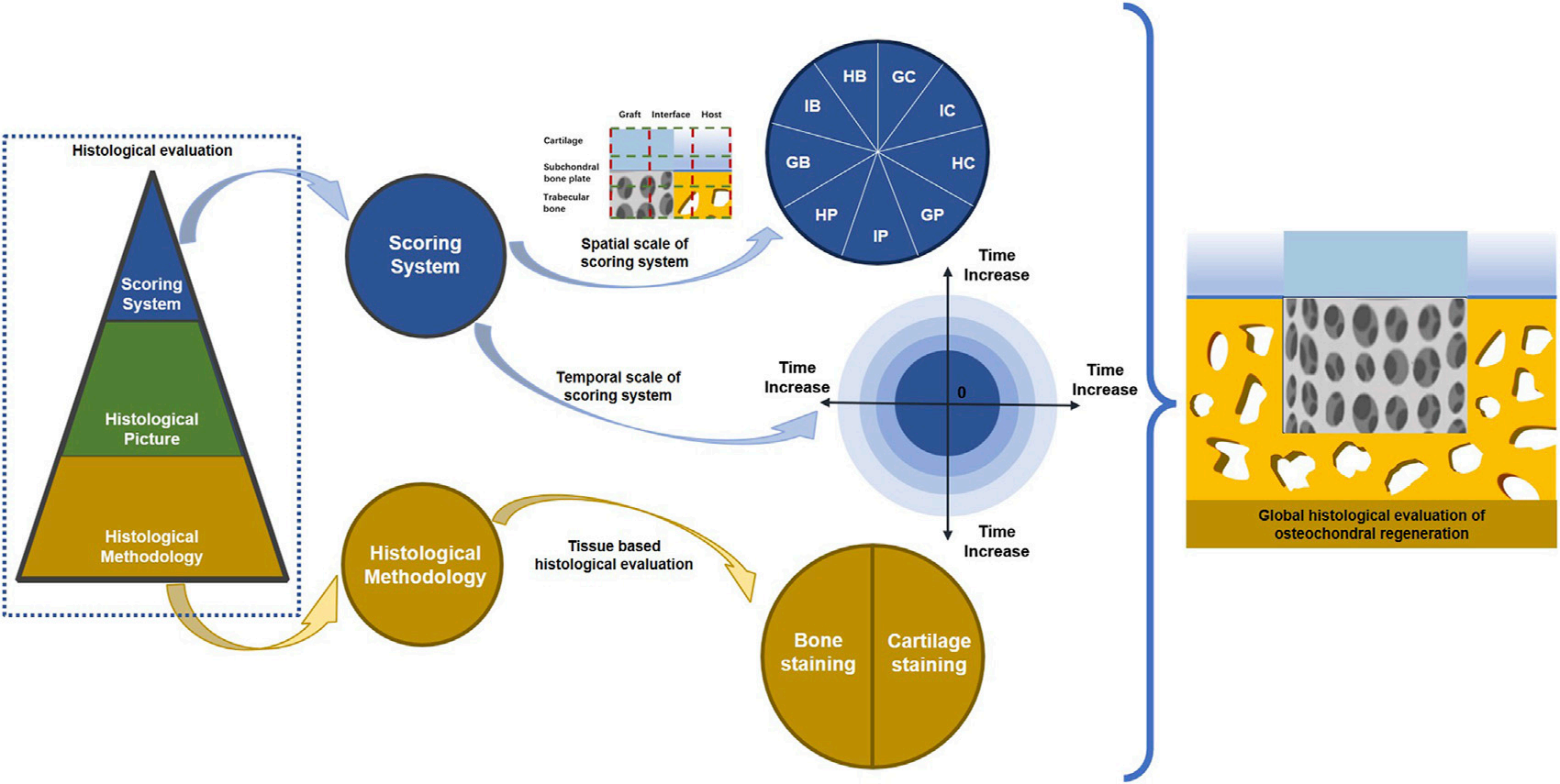
Potential improvement for current osteochondral regeneration evaluation. Global histological evaluation is recommended for osteochondral regeneration, which consists of spatial and temporal integrity for the scoring system. Furthermore, staining methods for cartilage and bone should be standardized for the histological scoring system.

Another limitation of current histological scoring systems for osteochondral repair is chronological inadequacy. Most studies usually select two timepoints to represent short-term and long-term outcomes separately (Gu et al., 2022). However, it may be incorrect to draw conclusions from single timepoints. A promising solution may be to summarize the morphology of regenerated tissue, especially of cartilage and the subchondral bone plate, from more timepoints. Some studies have already summarized histological classifications of cartilage and subchondral bone during osteochondral repair (Orth et al., 2012b; Qiu et al., 2003). Such classifications can enable the grouping of similarly repaired tissues. Collecting regenerated tissues with similar pathological changes into groups can reveal potential risk factors that cause poor regeneration (Figure 5).

There are a number of limitations inherent to osteochondral histological scoring systems. Regenerated tissue is displayed on histologically stained images, whereas histological scoring systems are textually described. Variations in understanding the textually described scoring criteria are inevitable between different observers, even though scoring systems have been validated to ensure their reliability and comparability. A single histological section may still be interpreted in different ways, even when the same textual criteria are used. To overcome this limitation, the ICRS II scoring system offers image-based criteria, which can be found on the internet (Mainil-Varlet et al., 2010).

High-quality images are fundamental for histological assessments and include appropriate staining methods for specific tissue components, appropriate magnification to detect details, complete regions of interest, and appropriate selection of histological sections that correspond with radiological evaluations. Common staining methods for osteochondral units have been discussed in our review. Selecting the appropriate stain can facilitate the analysis of the newly formed tissues’ quality. Certain scoring systems provide suggestions for selecting the appropriate staining technique (Gao et al., 2021). The Sellers scoring system requires Safranin O/Fast green for cartilage matrix staining (Katagiri et al., 2017), whereas the O’Driscoll scoring system merely suggests Safranin O staining for the same purpose (Lin et al., 2019). Conversely, the Fortier scoring system requires toluidine blue staining to evaluate cartilage-related parameters (Tschon et al., 2021). Additionally, numerous scoring systems do not indicate any specific staining method for histological evaluation. No consensus exists on whether a standard stain is necessary for histological scoring systems for osteochondral repair; however, this should be investigated in future studies to determine the effects of staining methods on histological results.

In addition to histological scoring systems, immunohistochemical analysis is another method to evaluate osteochondral regeneration (Browe et al., 2022). Immunohistochemical staining uses antibodies to indicate the presence of specific molecules. For a comprehensive evaluation of osteochondral unit regeneration and hyaline layer quality, collagen II was mainly used as a hyaline cartilage marker (Xia et al., 2022; Abedin et al., 2022). Conversely, collagen I, an undesired component during osteochondral repair, indicates fibrous cartilage and was used to determine the quality of cartilage by immunohistochemistry (Wang et al., 2020). Because of the increasingly recognized role of subchondral bone, studies have focused on collagen X (Li et al., 2020) and osteocalcin (Zhu et al., 2020) to detect cartilage calcification and bone growth and finally to provide a complete picture of osteochondral regeneration. Conversely, although aggrecan (Kondo et al., 2021) and Sox9 (Zhang et al., 2019) represent alternative markers for investigating cartilaginous tissue, few studies have analyzed them (Lamparelli et al., 2022a). This review reveals that current histological scoring systems do not include immunohistochemical staining. The potential reasons for this are explained below. First, most histological scoring systems were established before the wide use of immunohistochemical staining in osteochondral regeneration. Most scoring systems considered in this review were established before or around 2000, whereas immunohistochemical evaluations became widely used only afterwards (Rutgers et al., 2010). Second, the original design purpose of scoring systems may also impede the inclusion of immunohistochemical evaluation. Certain systems, such as OARSI and Mankin scores, were developed for osteoarthritis assessment, whereas other systems, such as ICRS and OSWESTRY, were developed for clinical purposes. Such original purposes for scoring systems make immunohistochemical evaluation non-essential. Third, some proteins of interest, which are present in the cell membrane and cell matrix, may limit quantitative assessments of immunohistochemical staining (Ming et al., 2018). However, researchers usually compare immunohistochemical results to histological results. Considering that methodological advancements have enabled the immunohistochemical detection of specific proteins, such new approaches should facilitate future investigations of osteochondral regeneration (Lamparelli et al., 2021). Accordingly, future scoring systems should include immunohistochemical staining for a comprehensive evaluation of osteochondral regeneration.

*In vivo* osteochondral regeneration is a pivotal process for tissue-engineered osteochondral grafts before clinical application. During this translational process, animal models first consist of smaller animals, such as rats (Ming et al., 2018), rabbits (Rastegar Adib et al., 2022), and dogs (Stefani et al., 2020), and then consist of larger animals, such as horses (Murata et al., 2022), goats (Kostešić et al., 2022), and pigs (Zlotnick et al., 2022). The need to assess models at the joint scale has complicated histological processing. Longer decalcification, paraffin embedding, and complex sample dissection are time-consuming yet inevitable (Favreau et al., 2020). Additionally, it is difficult to fully include larger osteochondral defects in one histological image, especially at higher magnifications (Bothe et al., 2019). As such, incomplete defect regions will omit important details and hinder interpretation. To minimize these drawbacks, samples should be cut with a detailed plan to prevent potential tissue loss. Graft–host interface indicators or graft contours should be provided on histological images to facilitate quick and accurate positioning. Another possible method to improve the evaluation of osteochondral repair in large animals is to analyze their gait (Ding et al., 2022). As gait parameters better represent the clinical situation, gait analysis may be a promising method to verify histological scoring systems for large-scale osteochondral defect models in the future.

The processing of samples, but not the results of sample staining, may be influenced by the animal species. Osteochondral units share structural and compositional similarities in synovial joints across different mammalian species (Mendes et al., 2020). Therefore, staining methods may remain consistent across different animal models. Nevertheless, small animals tend to receive higher histological scores than large animals owing to their relatively smaller joints. Thus, similar histological scores between small and large animals do not necessarily indicate similar osteochondral regeneration. As such, osteochondral tissue engineering should consider different animal models. To begin with, smaller animal models should be used for screening and preliminary evaluations. This should be followed by the use of larger animal models for preclinical assessments (Nie et al., 2019). Another potential explanation for inconsistent histological scoring criteria is the variability among staining methods (Lamparelli et al., 2022a). To address this, future histological scoring systems should specify staining methods that adequately convey compositional regeneration.

## 7 Conclusion

In this review, we reported the current framework of histological assessments of *in vivo* osteochondral repair with tissue-engineered grafts. Common staining methods were divided into two main categories for the chondral and subchondral bone part. Safranin O/Fast green is the most widely used staining method for the cartilage layer, whereas Gomori, Van Gieson, and Alizarin Red staining detect new bone formation. Moreover, appropriately displaying histological images with comprehensive details is crucial for interpreting the quality of osteochondral tissue. Finally, there is still no widely accepted histological scoring system for osteochondral regeneration. An ideal system should analyze both cartilage and bone regions equally. Furthermore, performing histological observations over longer periods of time may minimize discrepancies caused by defect size and animal models. Histological classification of repaired cartilage and subchondral bone plate is a promising method for predicting the long-term performance of osteochondral tissue-engineered grafts.

## Abbreviations

Col, collagen; ECM, extracellular matrix; EDTA, ethylenediaminetetraacetic acid; GAGs, glycosaminoglycans; ICRS, International Cartilage Repair Society; IHC, immunohistochemical; PRGs, proteoglycans.
